# Mesocolic hernia following retroperitoneal laparoscopic radical nephrectomy: A case report

**DOI:** 10.1016/j.ijscr.2019.07.040

**Published:** 2019-07-31

**Authors:** Naohiro Yoshida, Fumihiko Fujita, Kosuke Ueda, Suguru Ogata, Takahiro Shigaki, Takato Yomoda, Takafumi Ohchi, Tomoaki Mizobe, Tetsushi Kinugasa, Yoshito Akagi

**Affiliations:** a Department of Surgery, Kurume University School of Medicine, Asahi-machi 67, Kurume-shi, Fukuoka, 8300011, Japan; b Department of Urology, Kurume University School of Medicine, Asahi-machi 67, Kurume-shi, Fukuoka, 8300011, Japan

**Keywords:** Internal hernia, Laparoscopic nephrectomy, Mesocolon

## Abstract

**Introduction:**

Small bowel obstruction (SBO) caused by an internal hernia through a mesocolon after retroperitoneal laparoscopic nephrectomy (RLN) is rare.

**Presentation of case:**

A 66-year-old man who had undergone RLN with bladder cuff excision for a left renal pelvic cancer. After the surgery, he experienced SBO repeatedly. Contrast-enhanced computed tomography (CT) and gastrografin contract radiography through a long tube showed an internal hernia through the mesocolon to the retroperitoneal space where the resected left kidney had been located. We performed a subsequent surgery for the internal hernia. Postoperative course was uneventful and currently he has no recurrence of herniation 6 months post-operatively.

**Discussion:**

Mesenteric defects that cause an internal hernia can be created inadvertently during RLN when the colon is mobilized medially, and the kidney is being detached from retroperitoneum. The removal of a kidney leads to a potential retroperitoneal space to which small intestine can migrate. While there is no absolute necessity in mobilizing the colon during the retroperitoneal laparoscopic approach, there is still a risk of making mesocolic defects directly in the retroperitoneal space.

**Conclusion:**

We need to perform operations with sufficient anatomical knowledge of retroperitoneal fascia and careful surgical techniques. The critical thing to prevent an internal hernia following RLN is to close the mesenteric defects intraoperatively. It is also important to suspect an internal hernia and do proper examinations promptly when patients had the symptoms of SBO after nephrectomy.

## Highlights

• An internal hernia after retroperitoneal laparoscopic nephrectomy is rare.
• Retroperitoneal approach has the risk of making mesocolic defects directly.
• To prevent internal hernia, we should close the mesenteric defects intraoperatively.

## 1 Introduction

Laparoscopic radical nephrectomy has gained acceptance as the preferred radical surgical approach for renal pelvic cancer because it provides equivalent cancer control and lower complication rates compared with open surgery [1,2]. It is performed through either a retroperitoneal laparoscopic approach or a transperitoneal laparoscopic approach [3]. In recent years, retroperitoneal laparoscopic techniques have been increasingly used [4]. Bowel-related complications after retroperitoneal laparoscopic nephrectomy (RLN) are less than 1% and small bowel obstruction (SBO) is usually caused by adhesions [5]. SBO caused due to an internal hernia through a mesocolon is extremely rare [5]. Here, we present a rare case report of mesocolic hernia following RLN. The presented case has been reported in line with the SCARE criteria [6].

## 2 Presentation of case

**Fig. 1:**
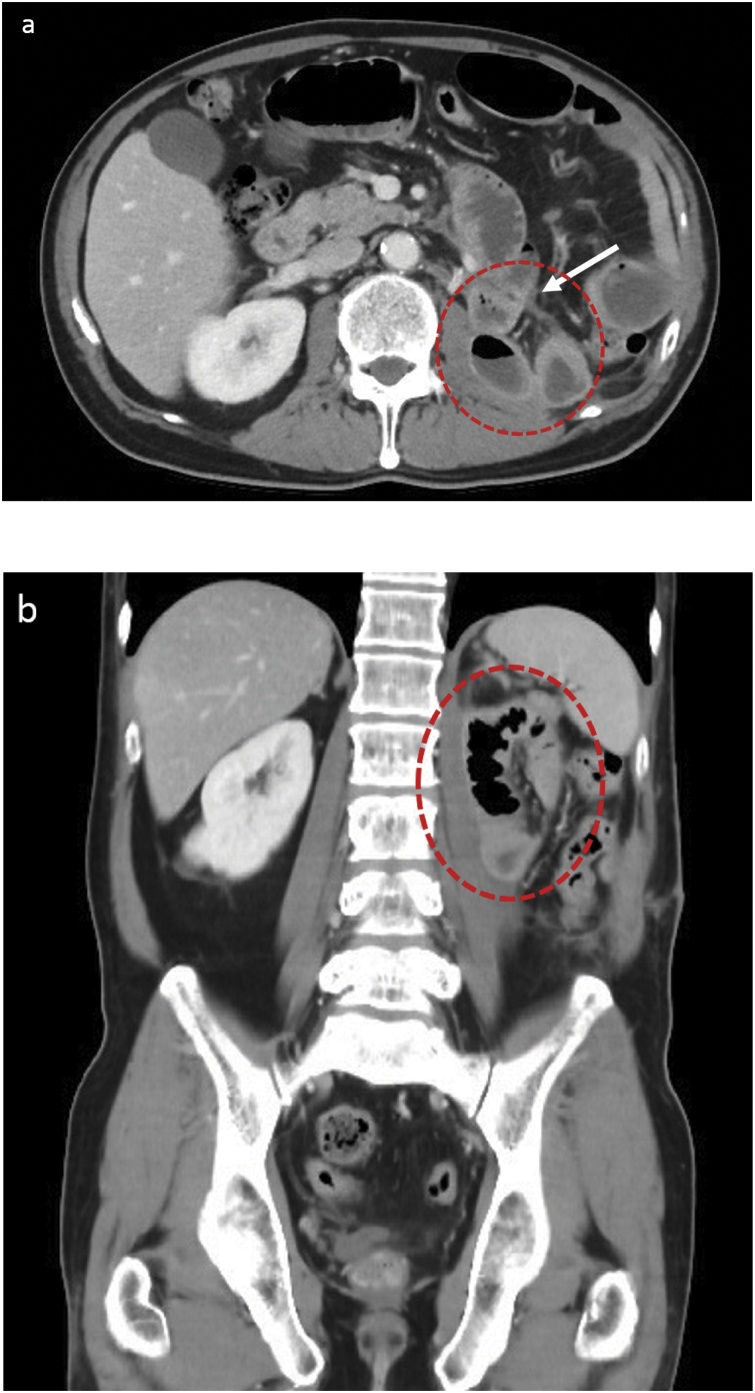
Enhanced computed tomography (CT) image showing the incarcerated small intestine in left retroperitoneal space. (dotted red circle: incarcerated small intestine, white arrow: the hernia orifice, a: horizontal image, b: coronal image).

**Fig. 2:**
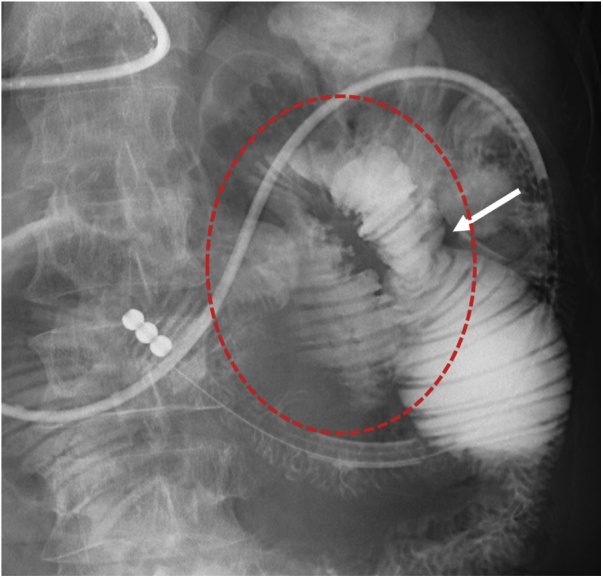
Gastrografin contrast radiography through a long tube showing the closed loop (dotted red circle) and caliber change of small intestine (white arrow).

**Fig. 3:**
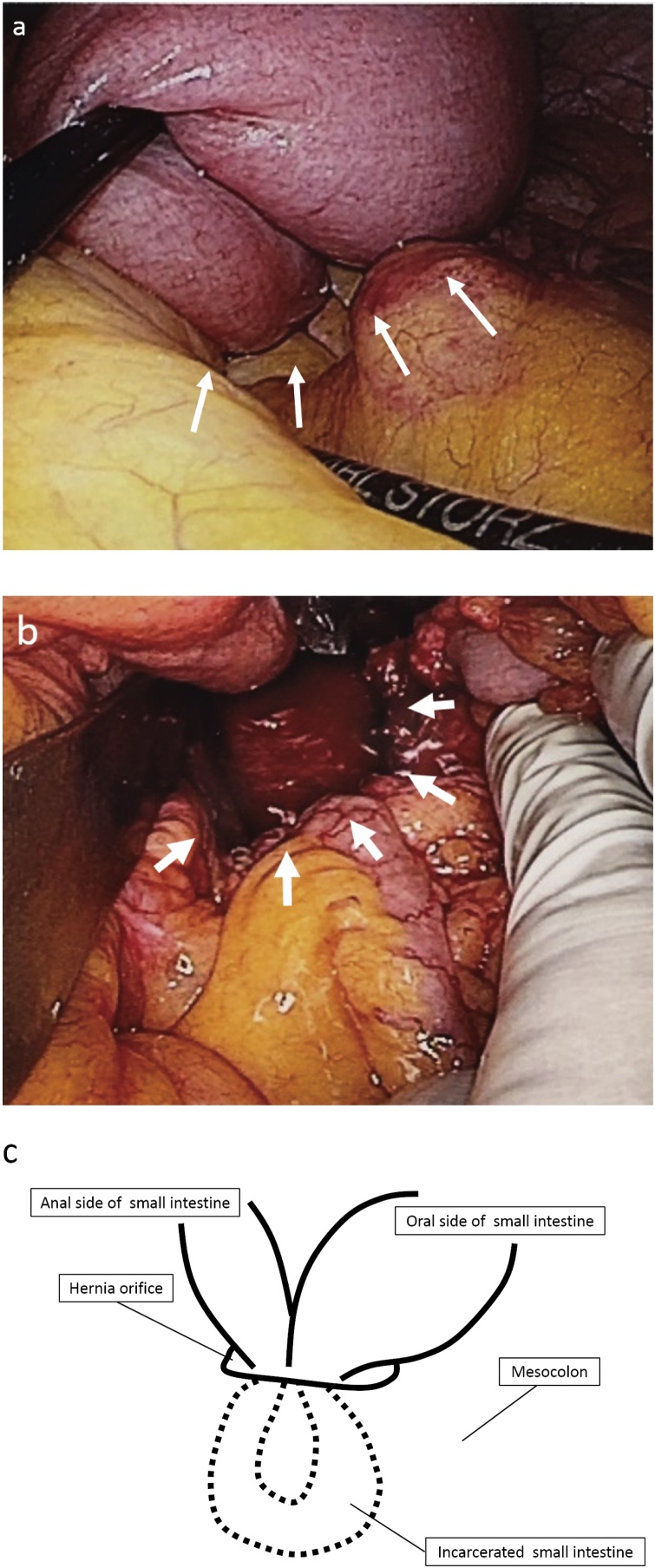
Intraoperative photographs and the schema of then internal herrnia. a: The incarceration of the small intestine through the mesocolon (white arrows). b: The hernia orifice cut to dilate after pulling the incarcerated small intestine (white arrows). c: The schema of the internal hernia.

The patient was a 66-year-old man who was diagnosed a left renal pelvic cancer, specifically invasive urothelial carcinoma, and underwent a RLN with bladder cuff excision in our hospital. One year ago, he had undergone a transurethral resection of bladder cancer and had been noted to have no other remarkable disease. RLN was performed with him in the right lateral decubitus position with access through three ports by the urology team. When they were clearing the perirenal fat, they made a tiny mesenteric defect from retroperitoneal space unintentionally. The defect was not closed during the operation because it was so small. After the isolation of the left kidney and the dissection of the renal vessels, they changed his position to a lithotomy position. They made a skin incision on the lateral edge of left rectus abdominis muscle, entered the retroperitoneal space then performed a total ureter resection and partial cystectomy. On postoperative day (POD) 1, he developed abdominal distension and vomiting. On the abdominal X-ray, he was diagnosed with SBO. A nasal gastric tube was inserted, and his symptoms improved temporarily but the SBO recurred three times. Computed tomography (CT) of the abdomen on POD17 revealed the upper small intestine was incarcerated into the retroperitoneal space resulting in a closed loop in the left upper quadrant of the abdomen through the left mesocolon. There were no ischemic findings (Fig. 1). Urologists consulted us and we inserted a long-tube and performed gastrografin contrast radiography through it. It demonstrated a closed loop of small bowel in the left upper quadrant, consistent with findings of CT scan (Fig. 2). We diagnosed SBO due to the internal hernia. As the status of the SBO had not improved, repeat surgery was performed. We started the operation using a laparoscopic approach. Intraoperatively, it was revealed that the small intestine was incarcerated into the retroperitoneal space through the mesocolon and the small intestine proximally was dilated (Fig. 3). The hernia orifice was 2 cm in diameter. We tried to free the incarcerated small intestine from the retroperitoneal space, but it was strongly adhered and difficult to exfoliate without injury. We converted the procedure to an open surgery. The hernia orifice was dilated carefully, and the incarcerated intestine was resected. It was difficult to close the hernia orifice of the mesocolon because its’ edge was strongly adhered to the retroperitoneum. We filled the hernia sac with omentum to prevent a recurrent intussusception of small intestine. Postoperative course was uneventful. The patient currently has no recurrence of herniation at 6 months post-operatively.

## 3 Discussion

Internal hernias are one of the rare causes of intestinal obstruction, comprising only 4% of all cases of SBO, and are caused by the defects within the peritoneal cavity [7,8]. Many types of internal hernias had been defined: paraduodenal, small bowel mesentery-related, greater omentum-related, and mesocolon-related [7,8]. They may be caused by congenital, postoperative or idiopathic reasons [8,9].

**Fig. 4:**
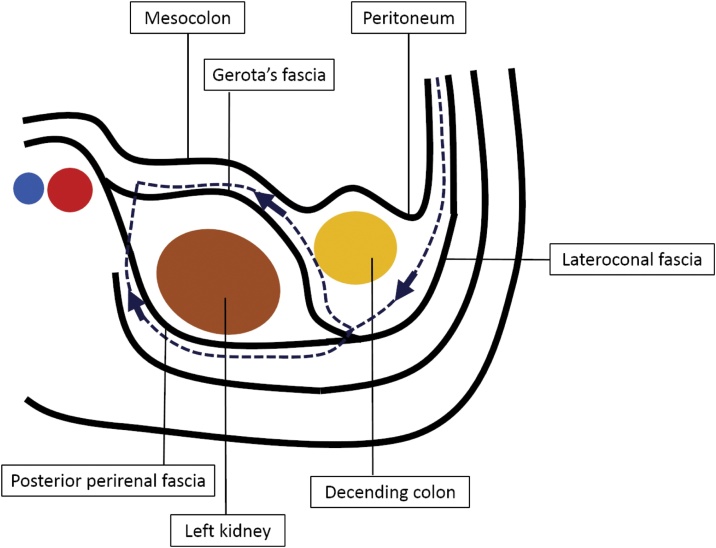
The schema of the retroperitoneal fascia and dissection line of radical RLN (blue dotted line with arrows).

We sometimes experience adhesional hernias after abdominal surgery, however, an internal hernia after RLN is rare. We searched PubMed for reports of an internal hernia after RLN, and only 17 cases have been reported in literature including our case (Table 1) [1,2,5,[10], [11], [12], [13], [14], [15], [16], [17], [18], [19]]. Radical nephrectomy for cancer and donor nephrectomy comprised of seven cases each. Reports of an internal hernia after radical nephrectomy make the majority of the cases in last 10 years. All the cases were on the left side. There are considered to be several reasons for an internal hernia after laparoscopic left nephrectomy. Mesenteric defects can be created inadvertently when the colon is mobilized medially as the lateral peritoneal reflection is incised and also when the kidney including Gerota’s fascia is being detached from retroperitoneum, and these then become the hernia orifices (Fig. 4) [10]. Another reason is the removal of the kidney leads to a potential space in the retroperitoneum to which the small intestine can migrate [1]. The significant dissection during laparoscopic radical nephrectomy for a malignancy can make larger potential space for an internal hernia than donor nephrectomy due to extensive colonic mobilization and mesenteric dissection for wide preparation of major vessels, and radial lymphadenectomy to obtain the appropriate cancer margins [1,5]. Kumar et al reported the attachment of the small bowel mesentery favors the disposition of the small intestine towards the mesentery of the descending colon under the influence of gravity and a defect in the mesentery of descending colon would be close to the proximal small intestine [3]. The most important thing to prevent an internal hernia following a nephrectomy is to recognize mesenteric defects and close them intraoperatively [3,13]. In our case, although we recognized a small defect in mesocolon, it was not closed because we had never previously experienced any problems from small defects after a nephrectomy.

**Table 1:** A summary of cases reported in literatures of internal hernia following laparoscopic nephrectomy.

No	Author	Year	Age(year)	Sex	Reason for nephrectomy	Location	Duration to reoperation from nephrectomy	Operative approach for reoperation	Operative procedure for reoperation
1	Knoepp [2]	1999	25	M	Donor nephrectomy	Left	6 weeks	Open Laparotomy	Reduction and repair
2	Regan [10]	2003	23	M	Donor nephrectomy	Left	5 days	Open Laparotomy	Reduction and repair
3	Regan [10]	2003	46	M	Donor nephrectomy	Left	1 week	Laparoscopy	Reduction and repair
4	Regan [10]	2003	59	M	Donor nephrectomy	Left	1 week	Open Laparotomy	Reduction and repair
5	Song [11]	2005	57	M	Urotherial carcinoma	Left	5 days	Open Laparotomy	Reduction and repair
6	Kocak [12]	2006	NA	NA	Donor nephrectomy	Left	NA	Laparoscopy	Reduction and repair
7	Letourneux [13]	2006	69	F	Atrophic kidney	Left	3 weeks	Open Laparotomy	Reduction and repair
8	Wong [5]	2008	43	M	Renal cell carcinoma	Left	11 days	Open Laparotomy	Reduction and repair
9	Cox [14]	2009	45	F	Oncocytoma	Left	9 weeks	Open Laparotomy	Reduction, resection and repair
10	Mehdi [15]	2009	62	M	Renal carcinoma	Left	4 weeks	Open Laparotomy	Reduction and repair
11	Leventhal [16]	2010	NA	NA	NA	NA	NA	Laparoscopy	Reduction and repair
12	Milosevic [17]	2011	55	M	Renal carcinoma	Left	3 weeks	Laparoscopy	Reduction and resection
13	Fitzgerland [18]	2013	67	F	Renal cell carcinoma	Left	13 months	Open Laparotomy	Reduction, resection and repair
14	Cuthbert [1]	2017	76	M	Renal cell carcinoma	Left	28 days	Open Laparotomy	Reduction, resection and repair
15	LaMattina [19]	2017	NA	NA	Donor nephrectomy	Left	NA	Laparoscopy	NA
16	LaMattina [19]	2017	NA	NA	Donor nephrectomy	Left	NA	Laparoscopy	NA
17	Our case	2019	66	M	Urotherial carcinoma	Left	23 days	Laparoscpy→Open Laparotomy	Reduction and resection

NA: not available.

Internal hernias can have mortality rates exceeding 50% if untreated, so early diagnosis is imperative in preventing intestinal ischemia [7,20]. However there are no specific symptoms of an internal hernia, so it is important to suspect the possibility of not only obstruction secondary to adhesions but also internal hernias when the patients present with the symptoms of SBO after nephrectomy. Serial abdominal examinations need to be promptly especially in cases of acute-onset or severe small bowel obstruction. Enhanced CT scan is the first-line imaging technique and very useful for the diagnosis of SBO. The insertion of a long-tube and gastrografin contrast radiography through it is useful too, for not only the diagnosis but also for the treatment of reducing the intraluminal pressure of dilated small bowel to perform a safe surgery [15].

The retroperitoneal laparoscopic approach has been increasingly used as more convenient and safer approach for nephrectomy because there is little interference from the abdominal organs. The limitation is that the working space is smaller than the transperitoneal laparoscopic approach [4]. In the retroperitoneal laparoscopic approach, the colon does not need to be mobilized in the way that is needed in the transperitoneal approach. However, there may be a higher risk of making mesocolic defects directly from the retroperitoneal space while detaching a kidney from the retroperitoneum in RLN.

## 4 Conclusion

It should be noted that there is a risk of making mesocolic defects directly while detaching a kidney from the retroperitoneum in RLN. We need to perform operations with sufficient anatomical knowledge of retroperitoneal fascia and careful surgical techniques. The critical thing to prevent internal hernia following RLN is to close the mesenteric defects intraoperatively when we recognized. It is also important to keep internal hernias higher on the differential and confirm this suspicion with prompt physical examinations when patients develop symptoms of SBO after nephrectomy.

Written informed consent was obtained from the patient for publication of this case report and accompanying images. A copy of the written consent is available for review by the Editor-in-Chief of this journal on request.

## Sources of funding

No funding.

## Ethical approval

Ethics Committee of Kurume University Scholl of Medicine, 21/6/2019, ref: No. 2019-015.

## Consent

Written informed consent was obtained from the patient for publication of this case report and accompanying images. A copy of the written consent is available for review by the Editor-in-Chief of this journal on request.

## Author contribution

NY contributed to conceptualization, study design, and manuscript drafting and editing. FF, KU and YA contributed to manuscript drafting and editing. SO, TS, TY, TO and TM contributed to data collection. FF, TK and YA contributed study supervision.

## Registration of research studies

Not applicable.

## Guarantor

Naohiro Yoshida, Fumihiko Fujita and Yoshito Akagi.

## Provenance and peer review

Not commissioned, externally peer-reviewed.

## Declaration of Competing Interest

All authors have no conflicts of interests.
